# Influence of sodium hypochlorite and EDTA on the microtensile bond strength of a self-etching adhesive system

**DOI:** 10.1590/S1678-77572010000400011

**Published:** 2010

**Authors:** Doglas CECCHIN, Ana Paula FARINA, Daniel GALAFASSI, João Vicente Baroni BARBIZAM, Silmara Aparecida Milori CORONA, Bruno CARLINI-JÚNIOR

**Affiliations:** 1 DDS, Graduate student, Endodontic Division, Department of Restorative Dentistry, Piracicaba Dental School, State University of Campinas, Piracicaba, SP, Brazil.; 2 DDS, Graduate student, Prosthodontics Division, Department of Restorative Dentistry, Piracicaba Dental School, State University of Campinas, Piracicaba, SP, Brazil.; 3 DDS, Graduate student, Department of Restorative Dentistry, Ribeirão Preto Dental School University of São Paulo, Ribeirão Preto, SP, Brazil.; 4 DDS, MS, PhD, Assistant Professor, Department of Restorative Dentistry, Dental School, University of Passo Fundo, Passo Fundo, RS, Brazil.; 5 Visiting Assistant Faculty, School of Dentistry University of California Los Angeles, CA, USA.; 6 DDS, MS, PhD, Associate Professor, Department of Restorative Dentistry, Dental School, University of São Paulo, Ribeirão Preto, SP, Brazil.

**Keywords:** Dentin-bonding agents, EDTA, Sodium hypochlorite

## Abstract

Chemical substances used during biomechanical preparation of root canals can alter the composition of dentin surface and affect the interaction with restorative materials.

**Objective:**

The purpose of this study was to evaluate the microtensile bond strength (µTBS) of a self-etching adhesive system to dentin irrigated with sodium hypochlorite (NaOCl) and ethylenediaminetetraacetic acid (eDTA).

**Material and Methods:**

Thirty human third molars were sectioned 3 mm below the occlusal surface, polished with 600- to 1200-grit silicon carbide papers, and randomly divided into 3 groups: G1 (control): no irrigating solution; G2: 1% NaOCl; and G3: 1% NaOCl followed by the application of 17% eDTA. The specimens received the self-etching adhesive system (XeNO III - Dentsply), restored with microhybrid composite resin (Z250 - 3M ESPE), sectioned and trimmed to create 4 hourglass-shaped slabs of each tooth. The slabs were tested in microtensile strength in a universal testing machine (emic DL 2000) at a crosshead speed of 0.5 mm/min until fracture. The results were analyzed statistically by ANOVA and Newman-Keuls test.

**Results:**

Mean µTBS values and standard deviations in MPa were: G1 = 11.89 ± 4.22; G2 = 19.41 ± 5.32; G3 = 11.34 ± 4.73. 1% NaOCl increased the adhesive resistance significantly (p<0.001/ F=22.5763). The application of 1% NaOCl/17% eDTA resulted in statistically similar µTBS to the control group.

**Conclusions:**

None of the irrigants affected negatively the µTBS of XeNO III to dentin. The use of 1% NaOCl alone resulted in higher bond strength than the other treatments. The combination of 1% NaOCl and 17% eDTA produced similar bond strength to that of untreated dentin.

## INTRODUCTION

Effective cleaning and shaping of root canals and adequate apical seal are essential to the success of endodontic treatment^33^, and the appropriate restoration of devitalized teeth is fundamental to prevent bacterial infiltration^3,12^. In addition, the purpose of restoring endodontically treated teeth is to reestablish their functionality and esthetics, and avoid fracture of the remaining dental structure^1^. Vire^31^ (1991) verified that 59.4% of the failures in root-filled teeth occur during re-establishment of the lost dental structure.

Chemical substances used during biomechanical preparation of root canals can alter the composition of dentin surface and affect the interaction with restorative materials^5,17,21^. Sodium hypochlorite (NaOCl) and ethylenediaminetetraacetic acid (EDTA) are substances usually used during the endodontic treatment^15,26,30^. NaOCl is an auxiliary irrigant used during root canal instrumentation to promote debridement, lubrication, disinfection, tissue dissolution, collagen layer removal and dentin dehydration^7,8^. EDTA is indicated as a final irrigating agent that produces dentin demineralization and provides an excellent cleaning of the canal walls, improving the penetration of chemical substances and promoting a more intimate contact of the filling material with the radicular dentin^14^. EDTA acts on the inorganic components of the smear layer, leading to decalcification of the peri- and intertubular dentin. It also covalently binds to metal ions and sequesters calcium ions present in hydroxyapatite dentin^4^.

Endodontically treated teeth with a sufficient amount of sound coronary structure should preferably be restored with composite resin by the direct technique, due to its capacity to bond to dentin and increase the fracture resistance of the remaining dental structure^11^. This process requires appropriate interaction of the adhesive system with the dentin substrate^29^. However, the irrigating substances frequently used during the endodontic treatment could interfere in the bond strength of the composite resin to dentin^21,32^.

Studies evaluating the bond strength of dentin after the application of irrigating solutions present different methodology from the usual clinical protocol^16,22,23,27,28,34^ as regard concentration, presentation form (gel or liquid), and time that the solutions remain in root canal^13^, hence hindering appropriate comparisons with real clinical condition. These authors evaluated the influence of irrigating solutions on dentin bond strength after etching to verify the efficacy of a deproteinization technique.

In the present study, the irrigating solutions were placed in contact with the dentin for a longer period to simulate a restoration placed after completion of endodontic treatment. The microtensile bond strength (µTBS) of a self-etching adhesive system to dentin irrigated with NaOCl alone or combined with eDTA was evaluated, testing the null hypothesis that endodontic irrigants (NaOCl and eDTA) do not affect the bond strength of the self-etching adhesive system to dentin.

## MATERIAL AND METHODS

### Experimental Design

The factor under study was the irrigating solution at three levels: G1: No irrigating solution (control); G2: 1% NaOCl (Natufarma Pharmacy, Passo Fundo, RS, Brazil); G3- 1% NaOCl followed by the application of 17% eDTA (Biodynamics. Ibiporã, PR, Brazil). The restorative system was XeNO III self-etching adhesive (Dentsply DeTrey; Konstanz, Germany) and Z250 composite resin (3M ESPE, St Paul, MN, USA). The experimental units consisted of slabs of human dentin randomly distributed into the three experimental groups (n=40). The response variable was µTBS evaluated in MPa.

### Selection of Teeth

Thirty sound freshly-extracted human third molars were used in this study. Teeth were stored 0.5% chloramine solution at 4°C for 48 h for disinfection. Next, the teeth were cleaned with pumice/water slurry in Robinson brushes (Microdont, Socorro, SP, Brazil) and analyzed under x10 magnifying glass (Carl Zeiss, Jena, Germany). The teeth were stored in distilled water at 4°C.

### µTBS Test

Each tooth was individually included in PVC cylinder (25-mm diameter and 20-mm height) (Tigre, São Paulo, SP, Brazil) using colorless autopolymerizing acrylic resin (Jet Clássico, São Paulo, SP, Brazil), so that the occlusal surface faced upwards. The teeth were sectioned 3-mm below the occlusal surface in a metallographic sectioning machine (Struers Minitom, Copenhagen, Denmark) and were polished (Struers Abramin, Copenhagen, Denmark) with silicon carbide papers (600- to 1200-grit) of successively finer grits. The samples were washed for 60 s and stored in distilled water at 4°C for 24 h.

The specimens were randomly distributed into the following groups: in group 1 (control), no irrigating solution was applied; in group 2, 1% NaOCl (5 mL) was applied to the dentin surface every 5 min for 1 h, simulating the time that NaOCl-based irrigants are usually left in the root canals during endodontic treatment under clinical conditions; in Group 3, 1% NaOCl was applied as described for Group 2, followed by a 5-min final rinse with 17% eDTA (5 mL), simulating the duration of the final flush with this chelating agent during endodontic treatment under clinical conditions. After dentin treatments, all specimens were washed with distilled water for 60 s.

In order to perform the adhesive technique in accordance with the manufacturer’s instructions, the dentin received two layers of XeNO III selfetching adhesive system using microbrush tips (Dentsply/DeTrey; Konstanz, Germany) followed by light curing for 20 s at 450 mW/cm^2^ of light intensity (Radii device; SDI, São Paulo, SP, Brazil).

After hybridization, three increments (~ 2 mm each) of Z250 composite resin (Shade A2) were applied on the dentin surface with a #½ spatula, reaching a total height of 6 mm. each increment was light cured for 20 s with the Radii device.

**Figure 1:**
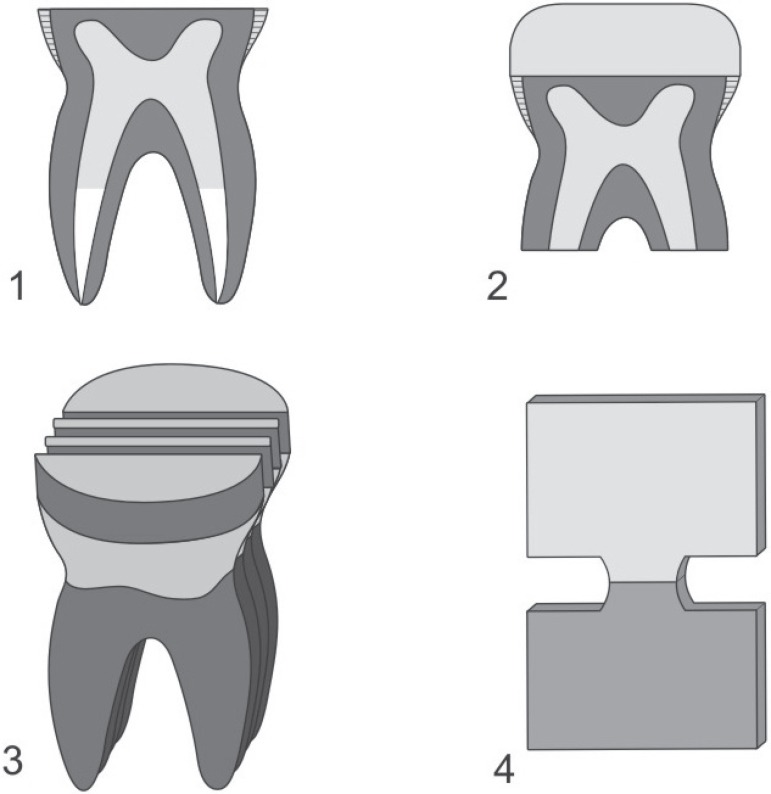
Schematic presentation of specimen preparation. (1) Exposed dentin, (2) Restored tooth, (3) Sectioning of the tooth to obtain four 1-mm-thick slabs from each tooth, (4) Hourglass-shaped specimen

After storage in distilled water for 24 h, the specimens were placed in a metallographic sectioning machine (Struers Miniton; Copenhagen, Denmark) and a water-cooled double-faced diamond disk was used to cut sequential longitudinal 1.0-mm-thick sections in a mesiodistal direction. Care was taken not to separate the slices. The specimens were then removed from the acrylic resin base through a transversal section, to obtain resin/dentin slabs measuring approximately 10-mm high, 5-mm wide and 1-mm thick. The slabs were trimmed on both sides of resin-dentin interface with a #1093 FF bur (KG Sorensen; Barueri, SP, Brazil) at a high-speed handpiece (Kavo; Joinvile, SC, Brazil) to obtain a 1-mm thick central area and produce standard hourglass-shaped specimens (Figure 1).

The specimens were individually fixed in a metallic device with a cyanocrylate adhesive (Loctite Super Bonder; São Paulo, SP, Brazil) so that the resin/dentin interface remained without any contact for the microtensile test. The metallic device coupled to a universal testing machine (Emic DL 2000; São José of Pinhais, PR, Brazil) and the specimens were subjected to a microtensile strength at a crosshead speed of 0.5 mm/min until fracture. At the moment of fracture, the resistance values were recorded in Newtons (N) by computer software.

Before the test, the area was measured with a digital caliper (Vonder Digital electronic Paquímetro; Curitiba, PR, Brazil) and the bond strength was calculated in MPa using the following equation: *Rt = F/A* , where *Rt* is the µTBS value, *F* is the force applied and *A* is the bond area between the dentin and restorative system. The data obtained were subjected to ANOVA and Newman Keuls tests (α=0.01).

## RESULTS

Table 1 shows the mean µTBS values and standard deviations (MPa) of the self-etching adhesive system to coronal dentin after the different treatments.

**Tabela 1:** Microtensile bond strength (pTBS) means ± standard deviations (MPa) of the self-etching adhesive system to coronal dentin after the different treatments

**Irrigating solution**	**μTBS**
	
No irrigating solution (G1)	11.89 ± 4.22 ª
1% NaOCI (G2)	19.41 ± 5.32 ^b^
1% NaOCI + EDTA 17% (G3)	11.34 ± 4.73 ª

Different letters indicate statistically significant difference (α=0.001)

There was statistically significant difference between the irrigating solutions. The use of 1% NaOCl alone resulted in higher µTBS of the selfetching adhesive system to dentin (p<0.001/ F=22.5763). There was no statistically significant difference between the use of 1% NaOCl combined with 17% eDTA and the untreated control group (Table 1), confirming the hypothesis under study.

## DISCUSSION

In the present study, the microtensile test was used due to the possibility of performing the analysis in an area of approximately 1.0-mm^14^, producing uniformity in the stress distribution and contributing to obtaining accurate results^18,20^. The microtensile test can be accomplished through the analysis of non-trimmed sticks or hourglass-shaped samples^20^. In this study, a small area was used to minimize potential defects and increase adhesion values^19^.

NaOCl is a halogenated compound, routinely used in endodontics, which has low surface tension, antiseptic ability, partially neutralizes the toxic products of root canals and dissolves organic tissue^2,14,24^. However, it does not act on the inorganic portion of dentin, which constitutes great part of the smear layer^9^. EDTA, according to Zaccaro, et al.^36^ (2010), presents a softening effect on dentinal walls that helps the instrumentation of the canals and has proven efficiency in the removal of the smear layer. The association of both substances is largely used in endodontic therapy because they act in organic and inorganic portion of dentin at the same time, hence making the instrumentation process more efficient^6,30,37^.

Considering that the adhesion of restorative materials to dentin of endodontically treated teeth can be altered^27^ when using irrigating solutions as NaOCl^32^ and eDTA followed by the application of the adhesive system^21^, and that higher adhesion values can be obtained using adhesive systems in dentin not previously treated by the irrigating solutions^35^, this study investigated whether self-etching adhesive system could also favor an increase in bond strength to dentin.

The outcomes of the present study revealed that irrigation with 1% NaOCl during 1 h (reapplied every 5 min) yielded higher µTBS of XeNO III selfetching system to dentin. A probable explanation for this fact is the superficial morphology of dentin treated with NaOCl, which does not remove the smear layer and expose the dentinal tubules^9^. The self-etching adhesive system has modified phosphoric acid on its composition (with high initial acidity) that incorporates the smear layer available on surface and forms the hybrid layer, increasing the bond resistance. Another factor that could justify the higher adhesion results can be related to the residual presence of water in the adhesive interface^25^.

Some studies showed that NaOCl affects the bond strength of adhesive materials to dentin, however, they present different methodologies from that used on this study, as regarding concentration, immersion time and presentation form of the irrigating solutions^13,16,27,34^, hindering appropriate comparison with the obtained results.

Ozturk and Özer^17^ (2004) compared the effect of 5% NaOCl on bond strength of of Clearfil SE Bond, Prompt L Pop, Scotchbond Multi Purpose and Prime Bond NT to pulp chamber lateral walls and verified significant decrease for all tested systems. Santos, et al.^22^ (2005) also observed adecrease in the bond strength of Single Bond after the deproteinization technique with NaOCl. Sauro, et al.^23^ (2009) found higher bond strength when NaOCl was applied on dentin after the acid conditioning and the authors emphasized that this technique may result in more durable resin-dentin bonds.

The application of the self-etching adhesive system promoted similar bond strength between the control group (no irrigating solution) and the group irrigated with 1% NaOCl followed by 17% eDTA, suggesting that the erosion in dentin surface caused by eDTA^4,10^ did not affect the formation of dentinresin bonds. Santos, et al.^21^ (2006) obtained lower bond strength values for a self-etching adhesive system when 5.25% NaOCl was used combined with 17% eDTA than the application of NaOCl alone on dentin.

Regarding the positive results of the self-etching adhesive system after treatment of dentin surface with irrigating solutions, further research should investigate the degradation of the adhesive/dentin interface formed with these systems after surface treatment with different root canal irrigants.

## CONCLUSION

Based on the obtained results and according to the employed methodology, it may be concluded that none of the endodontic irrigants affected negatively the µTBS of XeNO III self-etching adhesive system to dentin. While the use of 1% NaOCl alone resulted in higher bond strength than the other treatments, the combination of 1% NaOCl and 17% eDTA produced similar bond strength to that of untreated dentin.
